# Cellular heterogeneity and plasticity during NAFLD progression

**DOI:** 10.3389/fmolb.2023.1221669

**Published:** 2023-08-11

**Authors:** Hyun-Ju Park, Juyong Choi, Hyunmi Kim, Da-Yeon Yang, Tae Hyeon An, Eun-Woo Lee, Baek-Soo Han, Sang Chul Lee, Won Kon Kim, Kwang-Hee Bae, Kyoung-Jin Oh

**Affiliations:** ^1^ Metabolic Regulation Research Center, Korea Research Institute of Bioscience and Biotechnology (KRIBB), Daejeon, Republic of Korea; ^2^ Department of Functional Genomics, KRIBB School of Bioscience, University of Science and Technology (UST), Daejeon, Republic of Korea; ^3^ Biodefense Research Center, Korea Research Institute of Bioscience and Biotechnology (KRIBB), Daejeon, Republic of Korea

**Keywords:** NAFLD, heterogeneity, hepatocytes, Cholangiocytes, hepatic stellate cells, Kupffer cells, cellular plasticity, metabolic adaptation

## Abstract

Nonalcoholic fatty liver disease (NAFLD) is a progressive liver disease that can progress to nonalcoholic steatohepatitis (NASH), NASH-related cirrhosis, and hepatocellular carcinoma (HCC). NAFLD ranges from simple steatosis (or nonalcoholic fatty liver [NAFL]) to NASH as a progressive form of NAFL, which is characterized by steatosis, lobular inflammation, and hepatocellular ballooning with or without fibrosis. Because of the complex pathophysiological mechanism and the heterogeneity of NAFLD, including its wide spectrum of clinical and histological characteristics, no specific therapeutic drugs have been approved for NAFLD. The heterogeneity of NAFLD is closely associated with cellular plasticity, which describes the ability of cells to acquire new identities or change their phenotypes in response to environmental stimuli. The liver consists of parenchymal cells including hepatocytes and cholangiocytes and nonparenchymal cells including Kupffer cells, hepatic stellate cells, and endothelial cells, all of which have specialized functions. This heterogeneous cell population has cellular plasticity to adapt to environmental changes. During NAFLD progression, these cells can exert diverse and complex responses at multiple levels following exposure to a variety of stimuli, including fatty acids, inflammation, and oxidative stress. Therefore, this review provides insights into NAFLD heterogeneity by addressing the cellular plasticity and metabolic adaptation of hepatocytes, cholangiocytes, hepatic stellate cells, and Kupffer cells during NAFLD progression.

## 1 Introduction

Nonalcoholic fatty liver disease (NAFLD) is a prevalent liver disease, estimated to affect 25%−30% of the global population (Cotter and Rinella, 2020). It is defined as triglyceride accumulation resulting in more than 5% of hepatocytes containing lipid droplets without causes of alcohol consumption and viral infection (Semova and Biddinger, 2021). NAFLD is strongly associated with metabolic diseases such as obesity and type 2 diabetes (Kim et al., 2021). The prevalence of NAFLD parallels the increasing rates of obesity, as increased caloric intake, sedentary lifestyles, and the resulting obesity are major risk factors for NAFLD (Chaput et al., 2011; Byrne and Targher, 2015; Quek et al., 2023). Several studies also found that NAFLD is closely linked with an increased risk of type 2 diabetes, and the coexistence of NAFLD and type 2 diabetes accelerates the progression of NAFLD to nonalcoholic steatohepatitis (NASH), fibrosis, and hepatocellular carcinoma (HCC) (Budd and Cusi, 2020; Targher et al., 2021; Lee et al., 2023).

**FIGURE 1:**
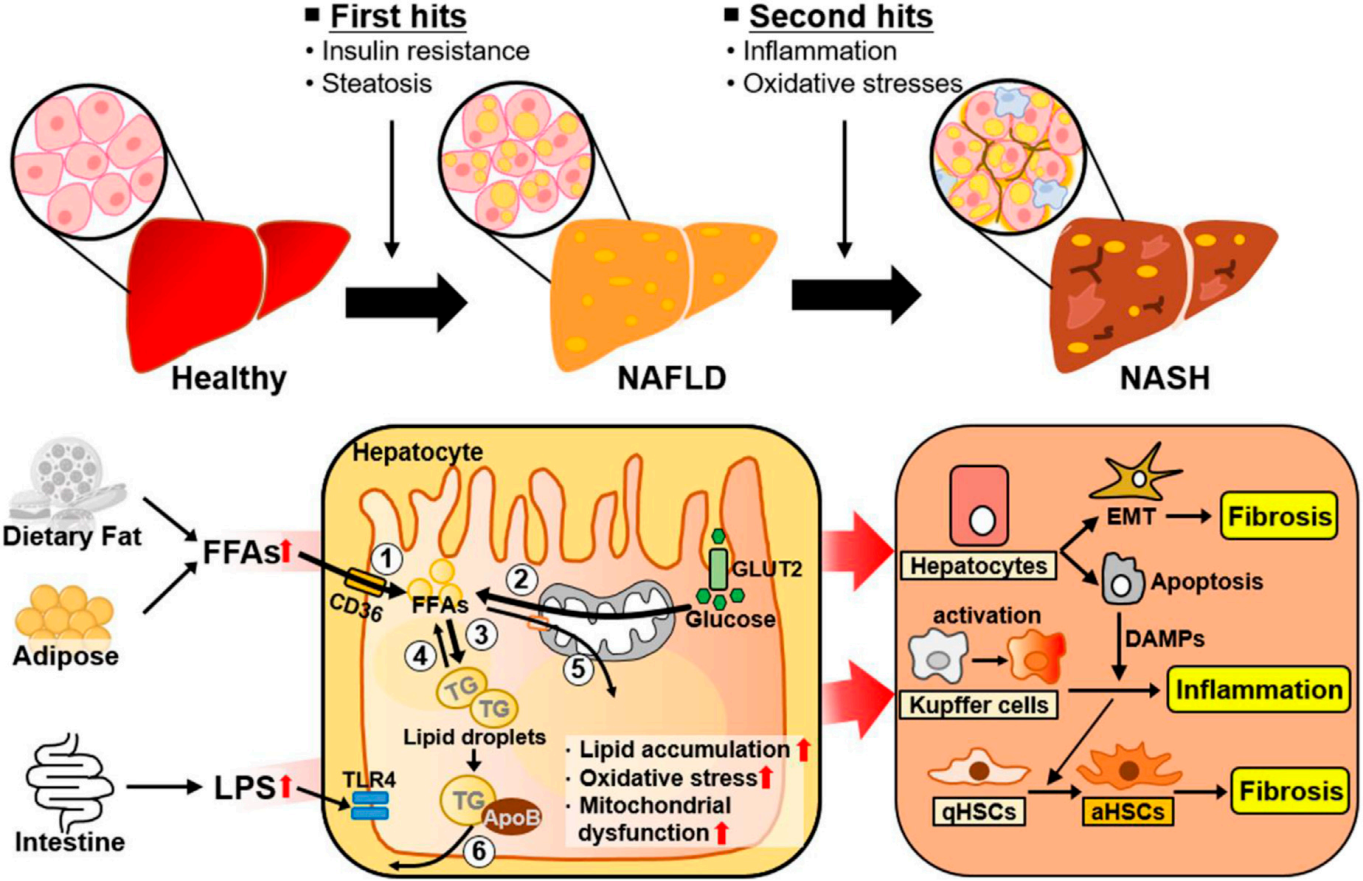
Metabolic changes and adaptive cellular plasticity induced by multiple factors during NAFLD progression. NAFLD is a complex disease influenced by a number of factors. The ‘first hit’ of NAFLD is excessive liver fat accumulation induced by insulin resistance. Hepatic lipid accumulation is closely associated with ^①^ increased hepatic FA uptake, ^②^ increased hepatic *de novo* FA synthesis, ^③^ increased lipogenesis, ^④^ decreased lipolysis, ^⑤^ decreased FA β-oxidation, and ^⑥^ decreased VLDL-TG secretion. The “second hit” of NAFLD is inflammation and oxidative stress. As well, NAFLD is developed and progressed by complex factors, such as endotoxins and other environmental factors. The liver consists of parenchymal hepatocytes and nonparenchymal cells (Kupffer cells, hepatic stellate cells, and endothelial cells), all of which have specialized functions. Transformation of NAFL to NASH is accelerated through cellular plasticity and adaptive metabolic changes regulated by the specific responses and crosstalk of these cells to various stimuli such as excessive intrahepatic FFAs, inflammation, and oxidative stress. Abbreviations: aHSCs, activated hepatic stellate cells; ApoB, apolipoprotein B; DAMPs, damage-associated molecular patterns; EMT, epithelial-to-mesenchymal transition; FFAs, free fatty acids; GLUT2, glucose transporter 2; LPS, lipopolysaccharide; NAFLD, nonalcoholic fatty liver disease; NASH, nonalcoholic steatohepatitis; qHSC, quiescent hepatic stellate cell; ROS, reactive oxygen species; TG, triglyceride; TLR4, Toll-like receptor 4.

The heterogeneity of NAFLD is driven by etiological complexity (Pal et al., 2021). NAFLD ranges from simple steatosis (or nonalcoholic fatty liver [NAFL]) to NASH as a progressive form of NAFL, which is characterized by steatosis, lobular inflammation, and hepatocellular ballooning with or without fibrosis (Pouwels et al., 2022). Traditionally, the progression of NAFL to NASH has been explained by the “two hit” hypothesis (Figure 1) (Dowman et al., 2010). The “first hit” is represented by abnormal hepatic fat accumulation attributable to excessive free fatty acids (FFAs) and insulin resistance. The “second hit” is inflammation and oxidative stress leading to liver injury in NAFLD. However, NAFLD is a complex disease influenced by multiple factors, and several molecular and metabolic changes induced by these factors can contribute to the phenotypic variability and heterogeneity of NAFLD (Figure 1). Reflecting the complexity and heterogeneity, the pathogenesis and progression of NAFLD have recently been described by the “multiple-hit” theory, which involves various factors, such as endotoxin, secretory factors, environmental and genetic factors, inflammation, and oxidative stress, acting in concert (Buzzetti et al., 2016; Tilg et al., 2021). Additionally, to further reflect current knowledge about this complex disease related with metabolic disorders, metabolic dysfunction-associated fatty liver disease (MAFLD) has also been introduced to describe fatty liver disease associated with metabolic disorders (Eslam et al., 2020; Fouad et al., 2020).

The underlying mechanism of the progression of NAFL to NASH can be described through an understanding of cellular plasticity (the ability of cells to transform from one phenotype to another without genetic alterations in response to environmental stimuli) and metabolic alterations in multiple liver cell types. The liver is a multifunctional organ with metabolic, biosynthetic, and detoxification functions. The liver cell population consists of two groups: parenchymal cells and nonparenchymal cells (NPCs) (Werner et al., 2015; Tian et al., 2023). Parenchymal cells, mainly hepatocytes, which are the primary effector cells for liver physiological and central metabolic function, account for approximately 80% of the total liver volume (Azparren-Angulo et al., 2021). Hepatocytes are metabolically active cells that function in glucose and lipid homeostasis, protein synthesis, bilirubin excretion, and detoxification. Cholangiocytes are biliary epithelial cells composing the hepatic parenchyma together with hepatocytes, although they account for only 3%–5% of the total liver mass. They play critical roles in bile production and composition, are variable and heterogeneous, and are closely related to chronic liver diseases. Hepatic NPCs comprise approximately 20% of the liver mass. NPCs are composed of liver sinusoidal endothelial cells (LSECs, approximately 40%), hepatic stellate cells (HSCs, 10%–25%), liver-specific macrophages known as Kupffer cells (approximately 30%), and other cell types (Su et al., 2021). NPCs are specialized cells that interact with parenchymal cells (hepatocytes) to form a functional hepatic unit. Several groups of cells support parenchymal cells in different ways. The transformation of NAFL to NASH is caused by the specific responses and crosstalk of these cells in response to various stimuli such as excessive intrahepatic FFAs, inflammation, and oxidative stress: Lipotoxicity and dysregulated apoptosis in hepatocytes (Geng et al., 2021); the release and activation of pro-inflammatory cytokines from Kupffer cells or adipokines from adipocytes (Duarte et al., 2015); and activation of HSC into collagen type I-producing myofibroblasts that form scar tissue (Higashi et al., 2017). The different responses of these cells are important factors contributing to the phenotypic heterogeneity of NAFLD.

This review aims to provide a better understanding of the metabolic alterations and phenotypic changes in hepatocytes, cholangiocytes, HSCs, and Kupffer cells in response to various metabolic stresses during NAFLD progression. We will provide insights into the heterogeneity and complexity of NAFLD by discussing the cellular and metabolic plasticity of these different cell types in the liver.

## 2 Parenchymal cells: Hepatocytes

**FIGURE 2:**
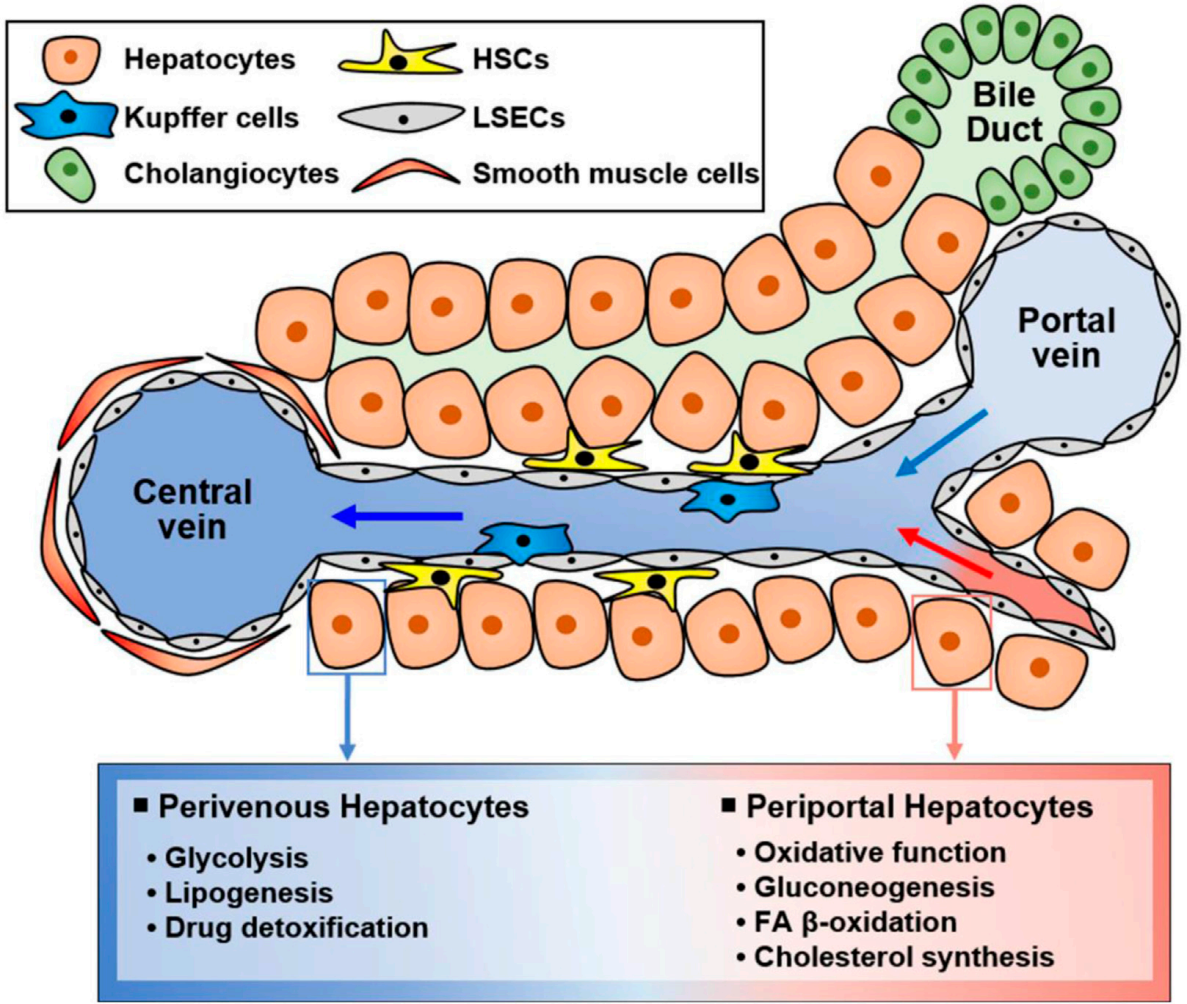
Functionally different hepatocyte populations according to their location in the liver. Liver cells are mainly composed of hepatocytes, hepatic stellate cells (HSCs), Kupffer cells, and liver sinusoidal endothelial cells (LSECs). Additionally, there are cholangiocytes in bile ducts and smooth muscle cells near the central vein. Liver cells have specialized functions depending on their location although hepatocytes are morphologically similar. Namely, hepatocytes located in the portal vein and central vein have different functions, suggesting that hepatocytes in different zones exert different functions. Periportal hepatocytes receive high levels of nutrients, oxygen, and hormones, and they are specialized for oxidative function, gluconeogenesis, FA β-oxidation, and cholesterol synthesis. Conversely, perivenous hepatocytes obtain low levels of nutrients, oxygen, and hormones and function in glycolysis, lipogenesis, and drug detoxification.

The liver is a multifunctional organ that is critical for metabolic homeostasis, and its functions include biosynthesis, detoxification, glycogen storage, and bile secretion (Trefts et al., 2017). Most liver functions are performed by hepatocytes (Trefts et al., 2017). Although all hepatocytes are morphologically similar, their functions are diverse depending on their location from the portal vein to the central vein (Figure 2). Hepatocytes located in the periportal and perivenous zones of the liver exhibit obvious differences in the levels and activities of enzymes and proteins (Trefts et al., 2017; Cunningham and Porat-Shliom, 2021). Periportal hepatocytes receive high levels of nutrients from the portal vein and oxygen and circulating hormones from the hepatic artery (Trefts et al., 2017; Cunningham and Porat-Shliom, 2021). Therefore, they are specialized for oxidative liver functions, making them critical for gluconeogenesis, fatty acid (FA) β-oxidation, and cholesterol synthesis. Conversely, perivenous hepatocytes are exposed to lower oxygen, nutrient, and hormone levels, and they function in glycolysis, lipogenesis, and drug detoxification. Therefore, hepatocytes can play various roles in response to various stimuli (Figure 2).

NAFLD is defined by the presence of lipid droplets in more than 5% of hepatocytes (Semova and Biddinger, 2021). It is characterized by hepatic steatosis and insulin resistance, which result from abnormal FA accumulation in the liver (Utzschneider and Kahn, 2006). These FAs mainly result from abnormal uptake driven by diet or adipose tissue lipolysis, and from increased hepatic *de novo* lipogenesis, via which FAs are newly synthesized from excess glucose (Kim et al., 2021). The six major mechanisms of lipid accumulation in the liver are 1) increased hepatic uptake of circulating FAs, 2) increased hepatic *de novo* FA synthesis, 3) increased lipogenesis, 4) reduced lipolysis, 5) decreased hepatic FA β-oxidation, and 6) decreased very-low-density lipoprotein (VLDL)-triglyceride (TG) secretion from the liver (Figure 1) (Kim et al., 2021). Namely, NAFLD arises when the amount of FAs in the liver accumulated through exogenous FA uptake and endogenous FA synthesis exceeds FA release (FA β-oxidation, lipolysis, and VLDL-TG secretion) from the liver (Ipsen et al., 2018; Kim et al., 2021).

**FIGURE 3:**
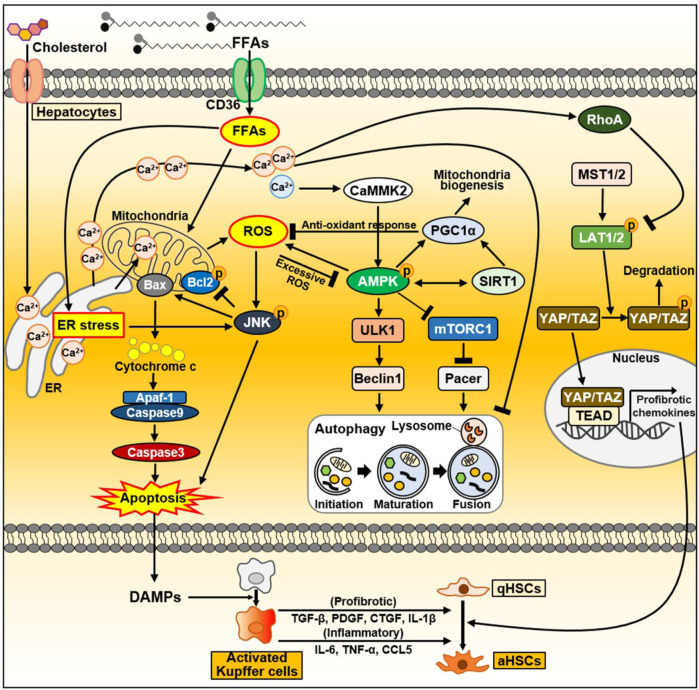
The signaling pathway in hepatocytes during NAFLD progression. FFAs are transported through CD36. FFAs enter cells via the FA transporter CD36. Excessive FAs cause mitochondrial dysfunction either directly or indirectly through ER–stress. Increased ROS production activates the JNK signaling pathway, which causes apoptosis through activation of the proapoptotic Bcl-2 protein Bax and suppression of the anti-apoptotic Bcl-2 family proteins. As a result, damaged or dying hepatocytes can release DAMPs to activate Kupffer cells. Activated Kupffer cells can secrete large amounts of inflammatory and profibrotic cytokines. Consequently, they can activate HSC, leading to liver fibrosis. In normal hepatocytes, increased ROS activate AMPK, which maintains intracellular homeostasis through PGC1α and SIRT1. Activated PGC1α can inhibit ROS production through anti-oxidant mechanisms. On the other hand, during NAFLD, FFAs accumulate intracellularly and can inhibit autophagy through AMPK inhibition and mTORC1 activation. In fatty liver, upregulated CD36 in hepatocytes inhibits autophagy initiation through the AMPK/ULK1/Beclin1 pathway. In addition, mTORC1 activation in NAFLD inactivates the autophagy enhancer Pacer, thereby interfering with autophagosome–lysosome fusion. As another key factor that can regulate autophagy flux, cytoplasmic Ca^2+^ activates AMPK through CaMMK2 in normal conditions. Conversely, excessively increased cytoplasmic Ca^2+^ during NAFLD disrupts autophagy flux. During NASH progression, YAP/TAZ in hepatocytes can be hyperactivated. Dietary cholesterol promotes ER-mediated Ca^2+^ secretion and activated Ca^2+^-RhoA pathway can sequentially stabilize TAZ. TAZ entering the nucleus strongly induces the expression of genes involved in HSC activation and fibrosis. Abbreviations: CCL5, C-C motif chemokine ligand 5; CTGF, connective tissue growth factor; IL-1β, interleukin-1β; PDGF, platelet-derived growth factor; TGF-β, transforming growth factor-β; TNF-α, tumor necrosis factor-α.

Excess lipid accumulation in hepatocytes leads to lipotoxicity, resulting in mitochondrial dysfunction associated with increased reactive oxygen species (ROS) levels, impaired autophagy, and altered extracellular vesicle and cytokine release. Dysfunctional organelles promote inflammation, and hepatocellular damage, eventually leading to hepatocyte apoptosis, through which damage-associated molecular patterns (DAMPs) are released (An et al., 2020; Wallace et al., 2022). When the liver is damaged, impaired cells are removed through inflammatory responses such as phagocytosis, and replaced by oval cells (Tanaka and Miyajima, 2016). During this process, a normal liver replaces dead epithelial cells with healthy epithelial cells, allowing it to return to its original structure and function (Tanaka and Miyajima, 2016). When liver injury occurs, hepatocytes undergo epithelial-to-mesenchymal transition (EMT), the change of epithelial cells to a mesenchymal phenotype (Zhao et al., 2016). Through the EMT process, hepatocytes acquire fibroblastic characteristics and progress to liver fibrosis (Choi and Diehl, 2019). In this section, we discussed intracellular signaling pathways and secreted factors in hepatocytes that can contribute to NAFLD progression and heterogeneity, and summarized in Figure 3.

### 2.1 Adaptive signaling and cellular plasticity in hepatocytes during NAFLD progression

#### 2.1.1 Lipotoxicity and lipoapoptosis

Liver cell apoptosis is a prominent feature associated with the severity of NASH (Feldstein et al., 2003). Apoptosis, a process of programmed cell death, is a method of cellular self-destruction that removes damaged cells to maintain homeostasis under both normal and pathophysiological conditions. In the pathogenesis of NASH, hepatocellular apoptosis is recognized as an important mechanism that can contribute to liver fibrosis. FFAs mediate lipoapoptosis, which is a potential mechanism of apoptosis associated with NASH (Malhi et al., 2006). Long-chain fatty acids (LCFAs) enter hepatocytes through FAT/CD36, originally characterized as an FA transport protein. These FAs can directly contribute to triacylglycerol accumulation in hepatocytes. FFAs can also enter the mitochondria and promote the activity of electron transport chain and FA oxidation, resulting in increased ROS production. Excessive ROS occur oxidative stress and alter cellular biomolecules (lipids, proteins, DNA), leading to mitochondrial dysfunction and liver injury (Evans et al., 2002; Cazanave and Gores, 2010). The c-Jun N-terminal kinase (JNK, also known as stress-activated protein kinase) signaling pathway is a key regulator in ROS-mediated cell death that well describes the relationship between elevated FFAs and lipoapoptosis as features of NAFLD (Malhi et al., 2006; Amir et al., 2012). FFAs can elevate ROS/oxidative stress and sequentially stimulate JNK activation. Activated JNK induces the lipoapoptosis of hepatocytes by stimulating the pro-apoptotic Bcl-2 protein Bax, which triggers the mitochondrial apoptosis pathway (Malhi et al., 2006). There are two major signaling pathways leading to apoptosis: the intrinsic apoptosis pathway initiated by mitochondrial events and the extrinsic apoptosis pathway initiated by death receptors such as tumor necrosis factor (TNF), TRAIL, and FAS-L (Dhanasekaran and Reddy, 2008). JNK can play a central role in both apoptosis pathways (Dhanasekaran and Reddy, 2008). The endoplasmic reticulum (ER) is also an organelle closely associated with lipotoxicity and lipoapoptosis (Cazanave and Gores, 2010). Both aberrant FFAs and FFA-induced ROS are critical factors that can trigger ER stress. Sustained ER-stress can activate the JNK signaling pathway and sequentially modulate the pro-apoptotic Bcl-2 family members, leading to hepatocyte apoptosis.

Conversely, AMP-activated protein kinase (AMPK), a mitochondria fine-tuning factor, can also be stimulated by ROS. Mitochondria-derived ROS acts on redox-sensitive cysteine residues (Cys-299/Cys-304) on the AMPKα subunit, and elevated ROS can activate AMPK by decreasing ATP levels or through S-glutathionylation of cysteine in the AMPKα and AMPKβ subunits (Filomeni et al., 2015; Hinchy et al., 2018). Activated AMPK occurs a PGC1α-dependent antioxidant response and lowers mitochondria ROS production, allowing for the maintenance of metabolic homeostasis and survival (Rabinovitch et al., 2017). AMPK exerts protective effects against hepatocyte lipotoxicity in the AMPK–PGC1α and AMPK–sirtuin 1 (SIRT1) axes (Li et al., 2020; Fang et al., 2022). Indeed, AMPK activity is reduced in NAFLD and NASH, and liver-specific AMPK reduction leads to NASH phenotypes such as fibrosis and cell death (Li et al., 2020; Fang et al., 2022). AMPK can ameliorate liver fat accumulation and NASH-associated hepatocyte apoptosis (Li et al., 2020; Fang et al., 2022).

DAMPs, also termed as alarmins, are endogenous risk factors that are released from damaged or dying cells to trigger strong inflammatory responses by activating the innate immune system via interactions with pattern recognition receptors (Bianchi, 2007; Roh and Sohn, 2018). Injured hepatocytes also release DAMPs, including metabolites, microRNAs, mitochondrial DNA, and mitochondrial double-stranded RNA (Kubes and Mehal, 2012). These DAMPs are recognized by macrophages, and they stimulate multiple inflammatory pathways through Toll-like receptors (TLRs) and inflammasomes (Zhang and Mosser, 2008; Schaefer, 2014). In NASH progression, hepatocyte-derived DAMPs promote the activation of Kupffer cells to remove damaged hepatocytes and accelerate liver inflammation (Kazankov et al., 2019). Activated Kupffer cells stimulate HSC activation by secreting inflammatory cytokines [TNFα, interleukin-1β (IL-1β), IL-6, C-C motif chemokine ligand 5 (CCL5)] and producing pro-fibrotic mediators (transforming growth factor β (TGF-β), platelet-derived growth factor [PDGF], connective tissue growth factor (CTGF)] (Wen et al., 2021). Additionally, HSCs have also a purinergic receptor P2Y14 as the DAMP receptor. The P2Y14 ligands uridine 5′-diphosphate (UDP)-glucose and UDP-galactose are abundant in hepatocytes. HSCs can be activated by P2Y14 ligands or secretory factors from dying hepatocytes in a P2Y14-dependent manner (Mederacke et al., 2022). Namely, DAMPs from damaged hepatocytes can promote inflammation and fibrosis by stimulating Kupffer cells and HSCs during NASH progression.

#### 2.1.2 Autophagy in hepatocytes

Autophagy is a self-degradative process and cellular recycling system that allows cells to degrade unnecessary or damaged organelles and pathogens to balance energy sources in response to environmental and nutrient stresses (Glick et al., 2010). Autophagic processes are directly linked with the development and progression of NAFLD. In the pathogenesis of NAFLD, the autophagy pathway mediates the degradation of intracellular lipids in hepatocytes, suggesting that the autophagy pathway is closely involved in the development of hepatic steatosis (Czaja, 2016). Simple steatosis (also known as NAFL) progresses to NASH, which is characterized by hepatocyte injury and death, inflammation, and fibrosis associated with increased oxidative stress and inflammatory cytokines (Browning and Horton, 2004; Chalasani et al., 2004). Autophagy regulates cell death signaling pathways induced by oxidants and TNF, which mediate NASH injury, and protects against cellular injury by removing damaged organelles in NASH (Czaja, 2016). NAFLD impairs autophagy, which is believed to involve various signaling pathways. Alterations in AMPK and mTORC1 activity can lead to impaired autophagy during NAFLD (Garcia et al., 2019; Li et al., 2019). Upregulation of CD36, a facilitator of membrane FA transport, inhibits autophagy initiation in the fatty liver by inhibiting the AMPK/uncoordinated 51-like kinase 1 (ULK1)/Beclin1 pathway. The negative effect of CD36 on autophagy is caused by the suppression of AMPK, a major activator of autophagy (Garcia et al., 2019; Li et al., 2019). Conversely, CD36 deficiency increases autophagy by activating AMPK, which enhances the activity of ULK1 and Beclin1that is important for autophagosome biogenesis (Mercer et al., 2018; Li et al., 2019). mTORC1 activation in NAFLD can impair autophagy by inhibiting ULK1/2 and inactivating the autophagy enhancer (Pacer) protein, thus interfering with autophagosome–lysosome fusion (Chen and Kilonsky, 2011; Carroll and Dunlop, 2017; He et al., 2020). mTORC1 activity might be influenced by AMPK (Carroll and Dunlop, 2017; He et al., 2020).

Intracellular Ca^2+^ homeostasis is important for regulating lipid and carbohydrate metabolism in normal hepatocytes (Ali et al., 2021). Chronic lipid exposure can alter Ca^2+^ homeostasis in hepatocytes (Ali et al., 2019; Ali et al., 2021). Lipids increase Ca^2+^ efflux from the ER and consequentially increase Ca^2+^ concentrations in the cytoplasm and mitochondria. Lipids can reduce lipolysis and β-oxidation and enhance lipogenesis, ER stress, ROS generation, and Ca^2+^/calmodulin-dependent kinase activation, resulting in the progression of NAFLD to more severe forms such as NASH, fibrosis, and HCC (Ali et al., 2019). Ca^2+^ is a key regulator of autophagy that acts through the regulation of pathways such as rapamycin complex 1, calcium/calmodulin-dependent protein kinase II, and protein kinase C signaling (Bootman et al., 2018). Ca^2+^ is also involved in autophagic signaling pathways including both mTOR and AMPK (Bootman et al., 2018). During obesity involving lipotoxicity, cytoplasmic Ca^2+^ levels are abnormally increased, and this is believed to attenuate autophagic flux by inhibiting autophagosome–lysosome fusion (Park et al., 2014). Obese mice treated with the calcium channel blocker verapamil exhibited rescued cytoplasmic Ca^2+^ levels in hepatocytes and impaired autophagosome–lysosome fusion (Park et al., 2014). These results indicate that altered intracellular Ca^2+^ levels can affect autophagy signaling during NAFLD progression.

#### 2.1.3 EMT in hepatocytes

EMT is the cellular process in which epithelial cells lose their apical–basal polarity and junction and acquire mesenchymal features (Kalluri and Neilson, 2003; Thiery et al., 2009). EMT contributes to organ fibrosis and promotes carcinoma progression (Thiery, 2002; Kalluri and Neilson, 2003; Thiery et al., 2009). EMT confers migratory and invasive properties to cells (Thiery et al., 2009). Because of cellular plasticity in the liver, epithelial cells can acquire fibroblastic properties through EMT, leading to fibrosis. Liver fibrosis is a protective reaction against chronic liver damage from multiple etiologies (Lotersztajn et al., 2005). It is characterized by the excessive deposition of extracellular matrix (ECM) produced by myofibroblasts (Miao et al., 2021). Activated HSCs (aHSCs) are considered the major origin of myofibroblasts (Iwaisako et al., 2012). However, hepatocytes can also undergo EMT to influence the fibroblastic phenotype during liver fibrosis (Zeisberg et al., 2007; Nitta et al., 2008). Hepatocytes stimulated with TGF-β1 display fibroblast-like morphology and express fibroblast-specific protein 1, which accounts for EMT in hepatocytes (Zeisberg et al., 2007). Other studies also showed that hepatocytes undergo EMT-like phenotypic changes in TGF-β-dependent manner and participate in fibrogenesis (Kaimori et al., 2007; Kojima et al., 2008). Conversely, specific inhibition of TGF-β signaling in hepatocytes delayed the fibrogenic phenotype (Dooley et al., 2008). The anti-fibrotic protein apamin can inhibit hepatic fibrogenesis by interfering with TGF-β1–induced EMT in hepatocyte (Lee et al., 2014). Celecoxib, a selective cyclooxygenase-2 (COX-2) inhibitor, ameliorates hepatic fibrosis by inhibiting hepatocyte EMT (Wen et al., 2014). These findings indicate that hepatocyte EMT contributes significantly to liver fibrosis.

#### 2.1.4 Hippo signaling pathway in hepatocytes

Hippo signaling plays an important role in the control of regeneration, stem cell self-renewal, and liver size (Pibiri and Simbula, 2022). It was recently reported that the Hippo signaling pathway is closely associated with fibrosis induction in multiple organs (Mia and Singh, 2022). Yes-associated protein (YAP) and transcriptional coactivator with PDZ-binding motif (TAZ), are the downstream effects of the mammalian Hippo pathway, and they function as transcriptional co-activators (Luo and Li, 2022). Recently, it was reported that YAP and TAZ in hepatocytes are hyperactivated during liver injury (Mia and Singh, 2022). YAP-expressing hepatocytes strongly induce genes related to inflammation (TNF, IL-1β) and fibrosis [collagen type I alpha 1 chain (COL1A1), tissue inhibitor of metalloproteinase 1 (TIMP-1), PDGF-C, TGF-β2] (Mia and Singh, 2022). Conversely, hepatocyte-specific deficiency of YAP and TAZ results in reduced inflammation, myoblast expansion, and liver fibrosis (Mia and Singh, 2022). Excessive cholesterol in hepatocytes can also promote liver fibrosis by upregulating TAZ through the Ca^2+^- RhoA signaling pathway (Wang et al., 2020). It has been reported that hepatocyte-targeted TAZ silencing in murine NASH models improves hepatic inflammation, and suppresses hepatocyte cell death and fibrosis (Wang et al., 2016). These results indicate that the Hippo signaling pathway in hepatocytes is involved in inflammatory and fibrotic processes during NAFLD/NASH progression.

## 3 Parenchymal cells: Cholangiocytes

In the liver, cholangiocytes and hepatocytes are the two main types of epithelial cells derived from hepatoblasts (embryonic liver stem cells) and are found in the liver parenchyma (Dianat et al., 2014; Huang et al., 2022; Sahoo et al., 2022). Cholangiocytes are biliary epithelial cells that line the bile ducts (Wang et al., 2022). They make up only 3%–5% of the total liver mass but play an important role in the production and homeostasis of bile, a digestive fluid that contributes to cholesterol excretion and detoxification in the liver (Boyer, 2013; Wang et al., 2022). Cholangiocytes also have liver regenerative potential (Sahoo et al., 2022). After liver injury, self-replication of hepatocytes is essential for maintaining liver size and function (Huang et al., 2022). However, when severe liver damage can destroy almost all hepatocytes, making hepatocyte regeneration impossible, hepatocytes are newly reproduced through the transdifferentiation of cholangiocytes (Sato et al., 2019; Huang et al., 2022). In other words, if hepatocyte regeneration is impaired by severe liver injury, cholangiocytes contribute to liver regeneration. Studies elucidating the pathogenesis of NAFLD in liver parenchymal cells have been mainly focused on hepatocytes (Lakhani et al., 2018; Lin et al., 2020; Lewis et al., 2023). However, the functional heterogeneity of cholangiocytes in the development and progression of NAFLD is important and is receiving increasing attention (Zhou et al., 2021; Cadamuro et al., 2022).

**FIGURE 4:**
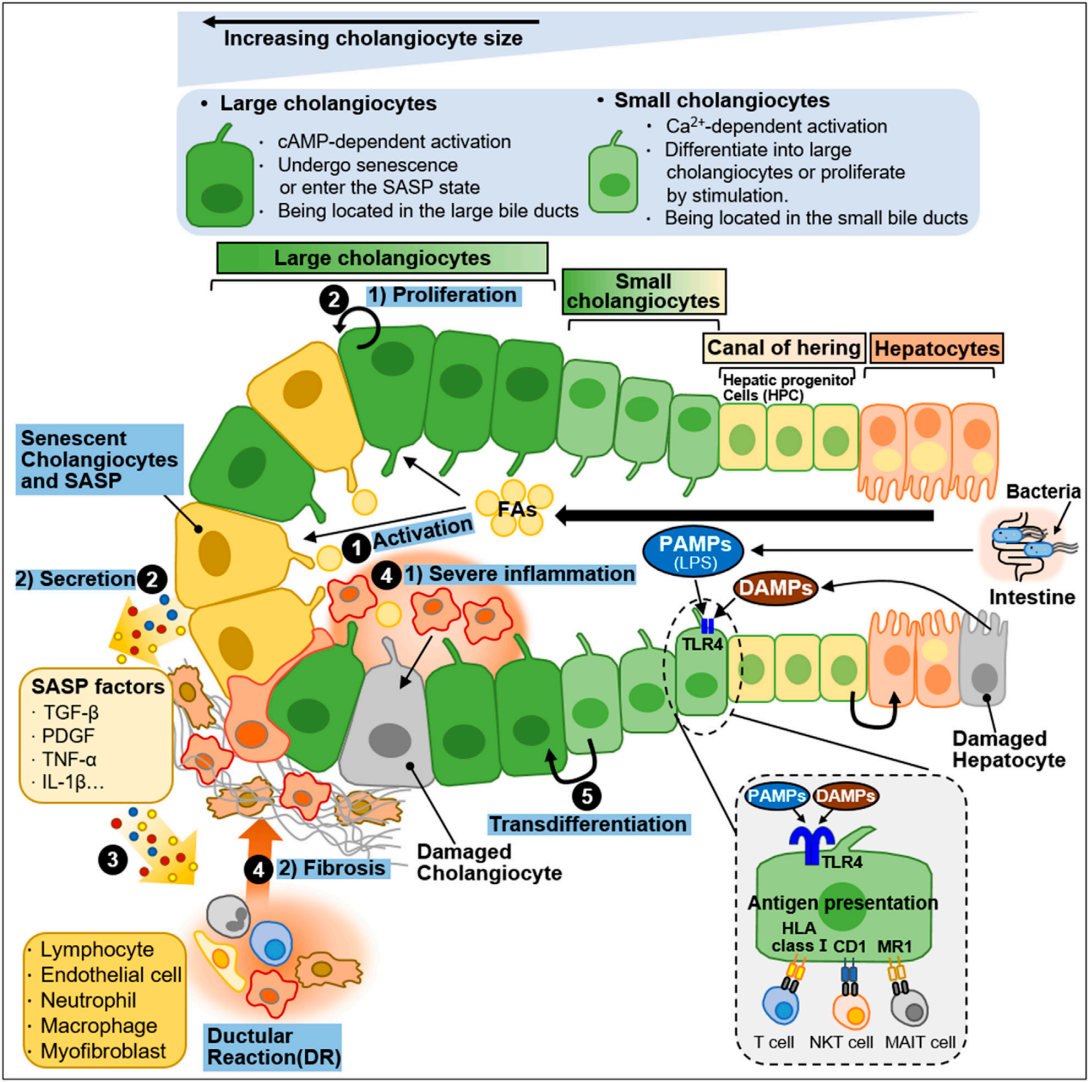
Heterogeneity of intrahepatic biliary epithelial cells during NAFLD progression. Ductal plate cells consist of cholangiocytes, canal of hering, and hepatocytes. Cholangiocytes are fundamentally divided into large cholangiocytes and small cholangiocytes based on the diameter of the bile duct (BD). Large cholangiocytes depend on cAMP-signaling and are more susceptible to damage than small cholangiocytes. During liver injury, large cholangiocytes enter a senescence-associated secretory phenotype (SASP) state and secrete proinflammatory factors that exacerbate the damage. When the large cholangiocytes are damaged, small cholangiocytes act as a progenitor to large cholangiocytes and differentiate into large cholangiocytes via Ca^2+^-activated signaling. In a healthy state, quiescent cholangiocytes play an important role in immune and antimicrobial defense in response to PAMPs (e.g., LPS) originating from the intestine or DAMPs derived from damaged hepatocytes. PAMPs and DAMPs bind to TLR4 expressed by biliary epithelial cells. Quiescent cholangiocytes can also present antigens to unconventional T-cells including NKT cells and MAIT cells. During NAFLD progression, activated cholangiocytes contribute to inflammation and fibosis. The process is as follows: ^①^ During NAFLD progression, excessive FAs stimulate cholangiocytes. ^②^ Activated cholangiocytes, also known as reactive ductular cells (RDCs), show 1) proliferation phenotype or undergo 2) senescence. Senescent RDCs secrete SASP factors (TGF-β, PDGF, TNF-α and IL-1β). ^③^ SASP factors stimulate immune cells and myofibroblasts, which promote inflammation and fibrosis, and some immune cells infiltrate into the bile duct. RDCs and mesenchymal and immune infiltration constitute ductular reaction (DR), a state in which they coexist. ^④^ The recruitment and infiltration of immune cells into the bile duct cause 1) severe inflammation and 2) fibrosis. ^⑤^ Small cholangiocytes transdifferentiate into large cholangiocytes to replenish the damaged cholangiocytes.

Cholangiocytes are heterogeneous in size and function and display active secretory functions that modify bile composition (Marzioni et al., 2002; Tabibian et al., 2013; Banales et al., 2019). Disruption of the homeostatic equilibrium in cholangiocytes is recognized by innate immune cells and can result in excessive deposit of scar tissue and biliary cirrhosis through inflammatory and pathological reparative reactions (Banales et al., 2019). In a heterogeneous response to environmental insults, some cholangiocytes exhibit reactive and proliferative phenotypes, whereas others display senescent and growth arrest phenotypes (Guicciardi et al., 2020). Both reactive and senescent populations of cholangiocytes significantly contribute to progressive liver failure, including inflammation and fibrosis, through the secretion of proinflammatory cytokines and chemokines (Guicciardi et al., 2020; Meadows et al., 2021). Ductular reaction (DR) and biliary senescence are hallmarks of cholangiopathies (Guicciardi et al., 2020; Meadows et al., 2021). They are increased in patients with NAFLD and NASH and are considered major causes of NAFLD progression (Sorrentino et al., 2005; Sato et al., 2019). In this section, we discussed the heterogeneity of cholangiocytes in NAFLD progression and summarized in Figure 4.

### 3.1 Cellular heterogeneity and plasticity in cholangiocytes during NAFLD progression

#### 3.1.1 Structural and functional properties of cholangiocytes

Cholangiocytes are morphologically and functionally heterogeneous (Glaser et al., 2009; Maroni et al., 2015). In humans, cholangiocytes are divided into small, medium, and large cells based on the correspondence with the diameter of the bile duct (BD) (Kanno et al., 2000; Glaser et al., 2009; Franchitto et al., 2013). In rodents, they exist in small and large sizes (Tabibian et al., 2013; Maroni et al., 2015; Little et al., 2023). Cholangiocytes exhibit different functions depending on their size. Small cholangiocytes line along the small intrahepatic BDs and have a relatively undifferentiated phenotype with large nuclei and small cytoplasm (Franchitto et al., 2013; Sato et al., 2018). They reside in the biliary progenitor cell compartment of the liver. They are known to be more resistant to damage than large cholangiocytes, and when large cholangiocytes are damaged by severe injury, they can differentiate into large cholangiocytes via Ca^2+^-activated signaling (Mancinelli et al., 2010; Mancinelli et al., 2013). Namely, small cholangiocytes can function as progenitor cells for large cholangiocytes. Large cholangiocytes line along the large intrahepatic and extrahepatic BDs and have small cytoplasm and large nuclei (Franchitto et al., 2013; Sato et al., 2018). Large cholangiocytes are thought to be more mature cholangiocytes than small cholangiocytes (Maroni et al., 2015). They are dependent on cyclic adenosine monophosphate (cAMP) signaling and are sensitive to injury (Glaser et al., 2009; Maroni et al., 2015). During liver injury, large cholangiocytes can undergo senescence and enter a senescence-associated secretory phenotype (SASP) state, secreting proinflammatory factors that exacerbate the damage (Huda et al., 2019). On the other hand, when the large cholangiocytes are damaged, small cholangiocytes proliferate and transdifferentiate into a large cholangiocyte phenotype, playing an important role in repairing the damaged epithelial lining (Mancinelli et al., 2013). These cell size-dependent differential responses will be thought to help understand the heterogeneity of cholangiocytes.

Under the healthy condition, quiescent cholangiocytes function as immunological and antimicrobial defense systems by secreting biliary immunoglobulin (especially secretory IgA) and various antimicrobial factors such as β-defensin 2, mucins, and lactoferrin into the bile (Farina et al., 2011; Demetris et al., 2016). These immunological functions are closely linked with the intestinal mucosal immune system (Chen et al., 2008; Demetris et al., 2016). Cholangiocytes that play an active role in immune pathogenesis fundamentally express TLRs (Chen et al., 2005; Chen et al., 2008). TLRs expressed by cholangiocytes recognize pathogen-associated molecular patterns (PAMPs) derived from the intestine or bloodstream, and consequently activated downstream signals induce the secretion of antimicrobial factors and inflammatory cytokines (Chen et al., 2005; Ninlawan et al., 2010; Oya et al., 2014). The complicated interaction between innate and adaptive immunity not only enhances biliary defense but also can promote inflammation. For example, intestine-derived lipopolysaccharide (LPS), a representative of typical PAMPs, and hepatocyte-derived DAMP can bind to TLR4 expressed by biliary epithelial cells during NAFLD development and progression (Guo and Firedman, 2010; Fabris et al., 2017). Persistent liver damage activates cholangiocytes to participate in liver inflammation by releasing inflammatory cytokines (Pinto et al., 2018).

Cholangiocytes are antigen-presenting cells that fundamentally express human leukocyte antigen (HLA) class I molecules (Syal et al., 2012; Schrumpf et al., 2015). Adhesion molecules such as HLA class II, leukocyte functioning antigen (LFA)-3, and intercellular adhesion molecule-1 (ICAM-1) are also expressed in cholangiocytes (Syal et al., 2012). Unconventional T cells are densely populated in sections adjacent to the liver and intestine (Godfrey et al., 2015). Cholangiocytes can present antigens to unconventional T cells including natural killer T (NKT) cells and mucosa-associated invariant T (MAIT) cells (Godfrey et al., 2015). Unconventional T-cell receptors recognize antigens expressed by HLA class I molecules (Mayassi et al., 2021). NKT cells perceive lipid antigens by a cluster of differentiation (CD) 1 by cholangiocytes (Schrumpf et al., 2015). MAIT cells perceive antigen bacterial B vitamins by MR1 molecules on cholangiocytes (Jeffery et al., 2016). Namely, cholangiocytes exposed to lipids activate NKT cells, and cholangiocytes exposed to bacteria activate MAIT cells, leading to releasing inflammatory cytokines and participating in inflammatory responses (Schrumpf et al., 2015; Jeffery et al., 2016).

#### 3.1.2 Activated cholangiocytes: Reactive and senescent cells

Cholangiocytes can be activated by various factors such as infection, endotoxins, and FAs (O’Hara et al., 2013; O'Hara et al., 2017). Activated cholangiocytes are characterized by increased proliferation and increased secretion of proinflammatory and profibrotic factors (Pinto et al., 2018; Strazzabosco et al., 2018). Activated cholangiocytes are involved in the inflammatory responses by releasing cytokines and chemokines (Adams, 1996; Chen et al., 2008). Sustained biliary cell damage can lead to excessive scar formation, biliary cirrhosis, and the chronic proliferation of cholangiocytes (Banales et al., 2019). Signals generated by the loss of cholangiocyte homeostasis drive the repair process (Fabris et al., 2016; Banales et al., 2019). These signals are recognized by inflammatory cells including macrophages and neutrophils and scaffold-producing cells including myofibroblasts and portal fibroblasts, and endothelial cells forming vasculature (Banales et al., 2019). These cells and corresponding signals form the biliary reparative complex referred to as ductular reactions (DR) (Sato et al., 2019; Chen et al., 2022). Namely, DRs are a response to a wide diverse of hepatobiliary damage that aims to recover compromised physical function after liver injuries (Jain and Clark, 2021).

Activated cholangiocytes, inflammatory cells, and mesenchymal cells are core cells that form DRs (Banales et al., 2019). As described above, activated cholangiocytes participate in inflammatory responses by secreting proinflammatory cytokines and chemokines. These secreted inflammatory factors activate and recruit immune cells. Liver mesenchymal cells are also activated by these inflammatory signals from the bile duct and are attracted to the bile duct (Cai et al., 2023). Activated mesenchymal cells secrete vesicles and soluble paracrine factors and stimulate cells in the bile (Banales et al., 2019; Azparren-Angulo et al., 2021). This epithelial-mesenchymal interaction demands the complementary expression of agonists and their corresponding receptors by epithelial and mesenchymal cells. TGF-β, PDGF, and monocyte chemoattractant protein-1 (MCP-1)/CCL2 are known to be released by reactive ductular cells (RDCs) and can activate myofibroblast (Kinnman et al., 2003; Kruglov et al., 2006; Fabris et al., 2017). RDCs exhibit a biliary phenotype as an epithelial component located around the portal space (Roskams et al., 2004). However, they can acquire morphologically and functionally mesenchymal properties (Fabris et al., 2016). This well describes the phenotypic plasticity of RDCs. RDCs exhibit upregulated EMT markers [S100A4, vimentin, matrix metalloproteinase 2 (MMP2)] and decreased epithelial markers (E-cadherin) in chronic cholangiopathies (Fabris and Strazzabosco, 2011). This ability of RDCs to increase mobility is essential for wound repair (Park, 2012). RDCs-induced biliary repair is mediated by morphogenetic pathways (Fabris et al., 2017). Among them, the Notch and YAP/TAZ pathways play critical roles in maintaining the biliary structure during biliary repair (Fiorotto et al., 2013; Morell et al., 2017; Panciera et al., 2017). Hepatocyte Notch activation induces hepatocyte-to-cholangiocyte conversion that expresses biliary SOX9 and HNF1β (Morell et al., 2017). Direct interaction of Notch-expressing hepatic progenitor cells (HPCs) with Jagged-1-expressing portal myofibroblasts occurs HPCs-to-RDCs conversion (Strazzabosco and Fabris, 2013; Nakano et al., 2017). Additionally, Notch signaling plays an important role in branching tubulogenesis in bile duct repair (Fiorotto et al., 2013). Depletion of Notch signaling attenuates biliary repair, whereas sustained Notch activation can lead to liver epithelial dysplasia and HCC (Geisler and Strazzabosco, 2015). These suggest that reactive cholangiocytes contribute to advanced liver failure including inflammation through secretion of proinflammatory cytokines and fibrosis through cholangiocyte proliferation and direct interaction with myofibroblasts.

Circulating FAs are increased in patients with NAFLD and NASH (Puri et al., 2009; Zhou et al., 2016; Feng et al., 2017). Excessive FAs induce cholangiocyte lipoapoptosis in a FoxO3/miR-34a-dependent manner. NAFLD patients show increased DR and fibrosis as markers of cholangiocyte damage (Natarajan et al., 2014; Natarajan et al., 2017). Cytokeratin 19 (CK19) is a biliary epithelial marker that is detected in the cytoplasm of DR-positive hepatobiliary cells (de Lima et al., 2008; Cai et al., 2016). CK19-positive DRs is more pronounced in areas with severe liver damage, as confirmed in liver sections from choline-deficient high-trans-fat diet-fed rat as a NAFLD model (de Lima et al., 2008). Biliary senescence is closely related to NAFLD and NASH progression (Meijnikman et al., 2021). Senescent RDCs can amplify inflammation and fibrotic responses through the senescence-associated secretory response (SASP) mechanism. Senescent cholangiocytes exhibit increased secretion of SASP factors such as TGF-β, PDGF, TNF-α, and IL-1β (Wu et al., 2016; Lopes-Paciencia et al., 2019; Blokland et al., 2020). This induces the recruitment and infiltration of immune cells and consequently exacerbates microvesicular steatosis and fibrosis during NAFLD progression.

## 4 NPCs: HSCs

HSCs are pericytes existing in the space between hepatocytes and LSECs, and they account for 5%–8% of the human liver (Lele et al., 2022). In healthy livers, most HSCs are quiescent and contain numerous cytoplasmic lipid droplets (Kamm and McCommis, 2022). The cytoplasmic lipid droplets in quiescent HSCs (qHSCs) play a major role in storing retinoids, a synthetic form of vitamin A (Bobowski-Gerard et al., 2018; Haaker et al., 2020). HSCs store approximately 80% of all retinoids in the body (Bobowski-Gerard et al., 2018; Haaker et al., 2020). Vitamin A (retinol) plays an important role in normal growth, reproduction, regeneration, immune responses, and intracellular metabolism. Upon liver injury, HSCs lose retinoid-containing lipid droplets to become myofibroblast-like cells producing ECM proteins such as collagen (Bobowski-Gerard et al., 2018; Trivedi et al., 2021). aHSCs are characterized by reduced intracelluar vitamin A storage and peroxisome proliferator-activated receptor *γ* expression (Trivedi et al., 2021). During HSC activation, retinyl ester levels within cells decrease together with triacylglycerol levels (Haaker et al., 2020). However, HSC proliferation, contractility, and chemotaxis increase with the expression of HSC activation-related proteins such as smooth muscle actin (α-SMA) and abundant ECM proteins such as fibronectin and collagens. Liver damage induced by high-cholesterol diet consumption (Teratani et al., 2012), virus infection (Wang et al., 2016; You et al., 2023), or immune-mediated injury (Xu et al., 2012) can transform qHSCs into aHSCs.

**FIGURE 5:**
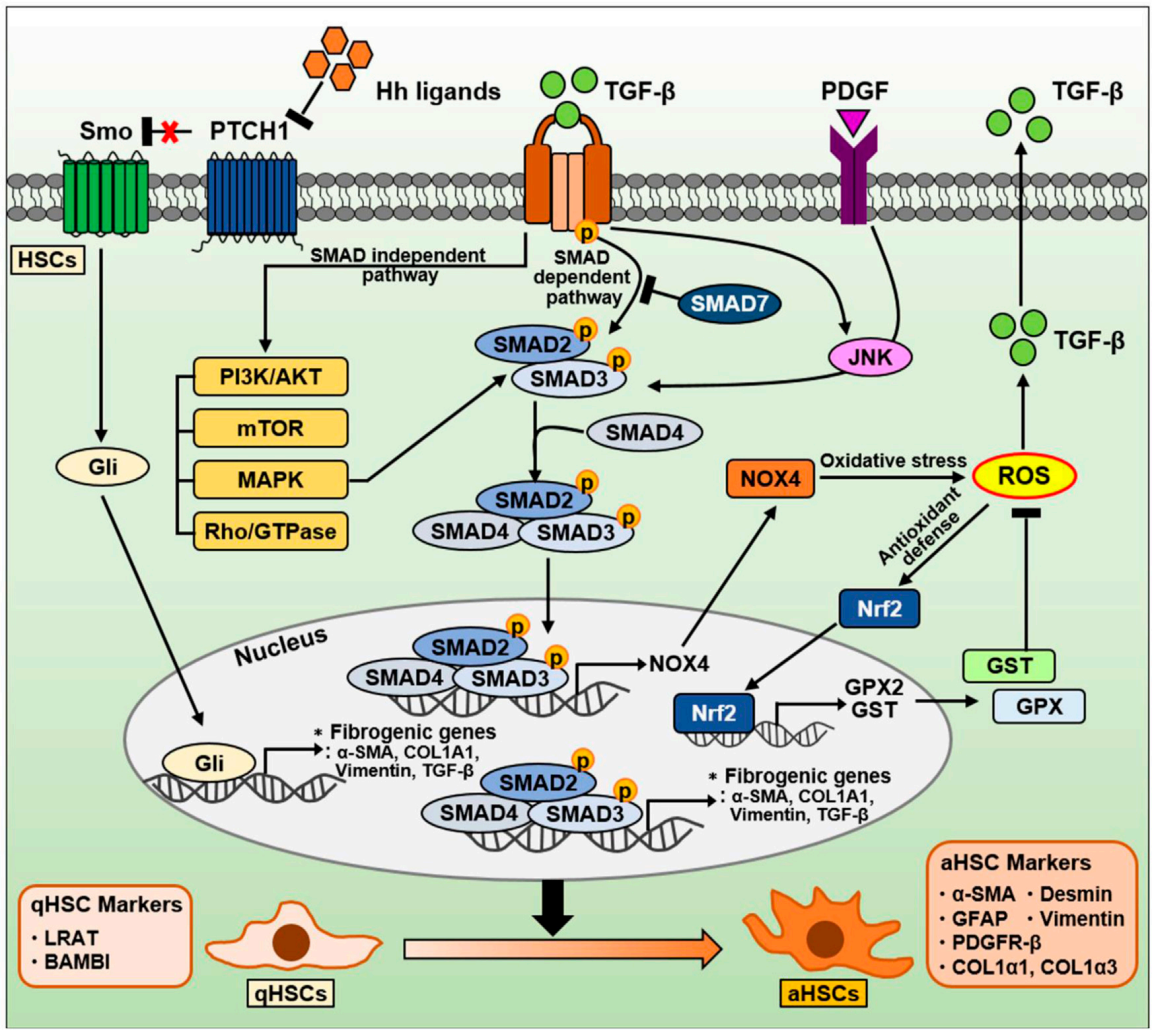
The signaling pathway in HSCs during NAFLD progression. TGF-β is well-known as a critical factor for HSC activation and liver fibrosis. The TGF-β signaling pathway is divided into a canonical SMAD-dependent pathway and a noncanonical SMAD-independent pathway. In a canonical SMAD-dependent pathway, TGF-β binds to and phosphorylates types I and II serine/threonine kinase receptors. Sequentially, it phosphorylates SMAD2/3 to form a complex with SMAD4. These SMAD complexes translocate into the nucleus, and regulates the expression of genes involved in oxidative stress and liver fibrosis. Both TGF-β and PDGF can also phosphorylate SMAD2/3 via JNK, leading to induction of fibrogenic genes. Conversely, anti-fibrotic SMAD7 can inhibit SMAD2/3 phosphorylation by directly binding to or indirectly degradation of TGF-β receptors. On the other hand, the SMAD2/3/4 complexes in nucleus can also induce NOX4, a major source of mitochondrial oxidative stress. NOX4-derived ROS production can increase TGF-β expression and secretion. Nrf2 is a transcription factor that mitigates ROS by increasing the expression of various ROS-detoxifying enzymes such as GPX2 and GST. On the other hand, TGF-β can also regulate various intracellular pathways, including PI3K/AKT, mTOR, MAPK, and Rho/GTPase, through a noncanonical SMAD-independent pathways. The Hh signaling pathway in HSCs is one of the important signaling pathways induced during NAFLD and NASH. During liver damage, Hh ligands are increased and then activate the Gli-responsive transcription factor to promote the expression of fibrogenic genes. Consequentially, qHSCs (qHSC markers: LRAT, BAMBI) transdifferentiate into aHSCs (aHSC markers: α-SMA, GFAP, PDGFR-β, COL1α1, COL1α3, desmin, and vimentin).

Liver fibrosis is a process that results in the excessive accumulation of ECM proteins, which can lead to the development of scar tissue (Bobowski-Gerard et al., 2018; Trivedi et al., 2021). Liver fibrosis can be caused by chronic liver injury such as NAFLD and NASH. aHSCs are the major sources of ECM proteins in the liver, producing large amounts of collagen and other ECM proteins that interfere with normal liver function. Researchers are currently investigating various strategies for targeting HSCs to prevent or reverse fibrosis in the liver, including the use of drugs that can inhibit HSC activation or promote their apoptosis (Ding et al., 2019; Kang et al., 2022). Therefore, understanding the role of HSCs in liver fibrosis is crucial for developing effective treatments for liver disease. In this section, we discussed the signaling pathways in HSCs during NAFLD progression and summarized in Figure 5.

### 4.1 Cellular plasticity in HSCs during NAFLD progression

#### 4.1.1 TGF-β/SMAD signaling pathways in HSCs

In the liver, TGF-β is an essential molecule for organ homeostasis that regulates organ size and growth by limiting cell proliferation and promoting apoptosis in hepatocytes (Giannelli et al., 2016; Fabregat and Caballero-Diaz, 2018). Loss of these functions can lead to hyperproliferative disorders including cancer (Dooley and ten Dijke, 2012; Giannelli et al., 2016). TGF-β exerts tumor-suppressive effects in the early stage of carcinoma, whereas it promotes carcinogenesis in the late stage of carcinoma (Massagué, 2008).

TGF-β is well-known as a key factor in HSC activation and ECM production, which triggers fibrosis in the liver (Fabregat and Caballero-Diaz, 2018). It contributes to the progression from initial liver injury, including inflammation and fibrosis, to cirrhosis and HCC (Dooley and ten Dijke, 2012). Cell plasticity can contribute to the adaptation of liver cells to metabolic stress and facilitate the transition from NAFL to NASH to fibrosis (Dooley and ten Dijke, 2012). During pathological conditions caused by chronic liver damage, HSC activation is mediated by various signals such as growth factors (e.g., PDGF, CTGF), lipids, ROS, cytokines produced by hepatocytes, Kupffer cells, endothelial cells, and cholangiocytes (Yin et al., 2013; Tsuchida and Friedman, 2017). Among these cytokines, TGF-β plays a central role in HSC activation from a quiescent state to an activated myofibroblastic phenotype (Dewidar et al., 2019). Namely, TGF-β is closely associated with the plasticity of HSCs, and it promotes liver fibrosis (Fabregat and Caballero-Diaz, 2018). At the plasma membrane level, TGF-β primarily binds to and activates types I and II serine/threonine kinase receptors to phosphorylate the downstream mediators SMAD proteins (Hata and Chen, 2016; Tzavlaki and Moustakas, 2020).

There are three different types of the SMAD proteins based on their functions: receptor-regulated SMADs (SMAD2, SMAD3, SMAD1/5/8), a common SMAD4, and inhibitory SMADs (Smad6, Smad7) (Hata and Chen, 2016). Among the SMAD proteins, TGF-β is known to upregulate SMAD2 and SMAD3, whereas downregulate SMAD7. The balance between SMAD2/3 and SMAD7 is known to play a critical role in liver fibrosis. Upon ligand binding, TGF-β receptors phosphorylate SMAD2/3, which forms a complex with SMAD4. This SMAD complex regulates the transcription of target genes including pro-fibrogenic genes along with cofactors after translocation to the nucleus (Hata and Chen, 2016). Conversely, the anti-fibrotic factor SMAD7 can directly bind to TGF-β receptors to inhibit SMAD2/3 phosphorylation (Yan et al., 2009), and can target and degrade Type I receptors by forming the complex with SMAD ubiquitination regulatory factor, an E3 ubiquitin ligase (Kavsak et al., 2000).

Several studies also describe SMAD3 as the major mediator of TGF-β–induced fibrogenic responses in HSCs, especially those associated with the induction of collagen expression (Inagaki et al., 2001; Schnabl et al., 2001; Furukawa et al., 2003). TGF-β can promote SMAD3 phosphorylation by activating p38 mitogen-activated protein kinase (MAPK), resulting in HSC activation and fibrosis progression (Inagaki et al., 2001; Schnabl et al., 2001; Furukawa et al., 2003). TGF-β and PDGF can activate HSCs via the JNK pathway (Yoshida et al., 2005). JNK in aHSCs directly phosphorylates SMAD2/3, resulting in the transcriptional activation of plasminogen activator inhibitor-1, which promotes HSC migration and fibrosis in liver tissue (Yoshida et al., 2005). Additionally, TGF-β can also activate noncanonical SMAD-independent pathways such as PI3K/AKT, mTOR, MAPK, and Rho/GTPase signaling pathways (Dewidar et al., 2019).

Meanwhile, SMAD7 can inhibit TGF-β–induced transdifferentiation and arrest HSCs in a quiescent stage by reducing the mRNA and protein levels of inhibitor of differentiation 1, which promotes hepatic fibrogenesis via the activin-like kinase 1/SMAD1 pathway (Dooley et al., 2003; Wiercinska et al., 2006). As well, miR-130a-3p can directly target TGF-β receptors and induce HSC inactivation and apoptosis, suggesting miR-130a-3p as a negative regulator in the progression of NASH through TGF-β/SMAD signaling (Wang et al., 2017).

#### 4.1.2 ROS-related signaling pathways in HSCs

ROS play a crucial role in the pathogenesis of liver fibrosis (Gandhi, 2012). They drive the activation of pro-fibrogenic HSCs, leading to ECM synthesis. During liver injury, ROS can be generated by two major enzyme families, namely, the NADPH oxidase (nicotinamide adenine dinucleotide phosphate oxidase [NOX]) and CYP450 families (Fabregat and Caballero-Diaz, 2018). Following liver injury, NOX isoforms in aHSCs are remarkably upregulated (Paik et al., 2011; Aoyama et al., 2012). For example, HSC-induced phagocytosis of hepatocyte-derived apoptotic bodies can activate NOX and stimulate TGF-β signaling and COL1A1 expression (Zhan et al., 2006; Jiang et al., 2010). NOX-generated ROS are also known to play essential roles in HSC activation and liver fibrosis (De Minicis and Brenner, 2007; Liang et al., 2016). ROS can stimulate TGF-β signaling by activating matrix metalloproteinases, enhancing TGF-β expression, or promoting TGF-β release (Richter and Kietzmann, 2016; Fabregat and Caballero-Diaz, 2018).

In an activated state of HSCs, NOX mediates pro-fibrogenic responses triggered by various stimuli, including TGF-β, PDGF, Ang II, and leptin (Liang et al., 2016). Among these stimuli, TGF-β is the most influential modulator that induces myofibroblastic HSC activation in the liver, leading to collagen protein and α-SMA expression (Gressner et al., 2002). The expression of NOX isoforms depends on the type of liver resident cells (Liang et al., 2016). Kupffer cells mainly express phagocytic NOX2, whereas hepatocytes, HSCs, and endothelial cells express phagocytic NOX2 and nonphagocytic NOX1 and NOX4. NOX4 mediates TGF-β-associated fibrogenic responses in various organs (Cucoranu et al., 2005; Hecker et al., 2009; Bondi et al., 2010; Sampson et al., 2011; Boudreau et al., 2012). In liver fibrosis, TGF-β enhances NOX4-induced ROS generation during HSC activation (Proell et al., 2007). In bile duct ligation (BDL)- and CCL4-induced fibrosis, TGF-β promotes NOX4 expression and activity via SMAD3 in HSCs (Jiang et al., 2012; Sancho et al., 2012).

Nuclear factor-erythroid 2-related factor 2 (Nrf2) is considered an antagonistic factor in liver fibrosis. As a transcription factor, Nrf2 modulate the expression of ROS-detoxifying enzymes such as glutathione S-transferase (GST) and glutathione peroxidase 2 (GPX2) (Tonelli et al., 2018). Nrf2 signaling represents a cytoprotective mechanism induced in cells exposed to oxidative stress (Tonelli et al., 2018). Further, Nrf2 exerts a protective effect against toxin-induced liver fibrosis (Xu et al., 2008). In line with this, sulforaphane, an Nrf2 activator, also improves hepatic fibrosis by inhibiting TGF-β signaling in the BDL model (Oh et al., 2012). Additionally, HSCs with reduced Nrf2 levels display markedly increased aHSC markers including ECM components through the TGF-β/SMAD signaling (Prestigiacomo and Suter-Dick, 2018). These well describe the association among TGF-β, ROS, and Nrf2 during the liver fibrosis process.

#### 4.1.3 Hedgehog (Hh) signaling pathways in HSCs

Hh pathway is a signaling cascade that regulates tissue morphogenesis by directing cell fate during embryogenesis and modulates injury-induced tissue remodeling in adults (Sicklick et al., 2005; Omenetti et al., 2011). It is a highly conserved and complex signaling pathway. The canonical Hh pathway consists of four main parts: 1) Hh ligands (Sonic hedgehog, Indian hedgehog, and Desert hedgehog), 2) cell surface receptor Patched (PTCH), 3) signal transducer Smoothened (SMO), and 4) glioblastoma (Gli) transcription factors as the effectors (Omenetti et al., 2011; Machado and Diehl, 2018).

HSCs participate in remodeling of the injured liver, and they can exert considerable plasticity during the fibrosis process. The Hh signaling pathway mediates adaptive responses during NAFLD, NASH, and fibrosis as well as in liver regeneration and injury (Fleig et al., 2007; Verdelho Machado and Diehl, 2016; Machado and Diehl, 2018). Hh ligands are rarely expressed in the healthy adult liver, whereas they are highly expressed during liver injury, activating the signal transduction pathway (Machado and Diehl, 2018). Hh signaling activation enhances the expression of fibrogenic genes (α-SMA, COL1A1, vimentin, and TGF-β) and Snail, a Gli-responsive transcription factor that mediates TGF-β–induced EMT, whereas it reduces the expression of qHSC markers (Choi et al., 2009). During HSC activation, bmp7 and its target id2 are downregulated, resulting in decreased E-cadherin expression (Valcourt et al., 2005). Conversely, inhibition of the Hh signaling recovers the expression of the epithelial markers (bmp7, id2, E-cadherin) and qHSC markers, and results in a loss of the fibrotic phenotype of aHSCs (Choi et al., 2009). EMT in HSC is closely associated with cytoskeletal reorganization. Therefore, it is accompanied by alterations in the activity of the small GTPase Rac1 that is associated with cytoskeleton. In both *in vitro* HSCs and *in vivo* mouse models, Rac1 activation promotes Hh signaling and the fibrogenic phenotype and aggravates liver fibrosis (Choi et al., 2010).

### 4.2 Cellular plasticity markers in HSCs

#### 4.2.1 qHSC markers

##### 4.2.1.1 Retinol processing proteins: Lecithin retinol acyltransferase (LRAT)

Most of the vitamin A in the body of healthy vertebrates is contained within lipid droplets present in the cytoplasm of HSCs (Bobowski-Gerard et al., 2018; Haaker et al., 2020). LRAT is an enzyme that catalyzes vitamin A esterification and is essential for maintaining vitamin A homeostasis (Nagatsumaet al., 2009; Blaner et al., 2009). LRAT is predominantly expressed in quiescent HSCs under healthy conditions in which HSCs have a lipid-storing phenotype (Nagatsumaet al., 2009; Shang et al., 2018).

##### 4.2.1.2 BMP and activin membrane-bound inhibitor (BAMBI)

The TGF-β–Pseudoreceptor BAMBI is a transmembrane glycoprotein that negatively regulates TGF signaling (Onichtchouk et al., 1999). It is mainly expressed in HSCs. It is highly expressed in qHSCs whereas low expressed in aHSCs (Liu et al., 2014).

#### 4.2.2 aHSC markers

##### 4.2.2.1 Cytoskeletal protein: α-SMA

α-SMA is considered a reliable marker of aHSCs and liver fibrosis (Carpino et al., 2005; Friedman, 2008; Hoffmann et al., 2020). This cytoskeletal protein is undetectable in normal liver. It exists exclusively in portal myoblasts and vascular smooth muscle cells but not in other liver cells (Nagatsuma et al., 2009; Shang et al., 2018). Its expression is used to quantify liver fibrosis in preclinical and clinical studies.

##### 4.2.2.2 Neural marker: Glial fibrillary acidic protein (GFAP)

GFAP is an intermediate filament protein found predominantly in astrocytes of the central nervous system (Hol and Pekny, 2015). GFAP is also expressed in the quiescent state of rodent HSCs (Geerts et al., 2001). GFAP expression has been reported to be absent in the normal human liver or observed in a small subpopulation of periportal cells (Zakaria et al., 2010; Hassan et al., 2014). In fibrotic livers, GFAP was detected in HSCs and the periseptal region of the regenerative nodules (Levy et al., 1999). It is considered a useful marker to validate early HSC activation (Hassan et al., 2014).

##### 4.2.2.3 ECM protein: COL1α1 and COL3α1

ECM proteins, such as collagens, fibronectins, and laminins, play critical roles in cell adhesion, migration, and tissue formation (Zhang et al., 2021). During liver injury, aHSCs undergo myofibroblastic differentiation that abundantly secrete ECM proteins (Zhang et al., 2016).

##### 4.2.2.4 Platelet-derived growth factor receptor beta (PDGFR-β)

PDGFR-β is a receptor tyrosine kinase that drives cellular fibrosis and proliferation (Bedekovics et al., 2013). PDGFR-β induction is a hall marker of HSC activation (Kocabayoglu et al., 2016). Its expression is extremely decreased in the healthy liver but markedly increased during liver injury (Breitkopf et al., 2005).

##### 4.2.2.5 Cytoskeletal protein: Desmin

Desmin is an essential intermediate protein that plays a critical role in maintaining the integrity and mechanical stability of HSCs (Geerts et al., 2001). It is one of the useful markers for identifying HSCs, and it is strongly upregulated during HSC activation (Zhang et al., 2018).

##### 4.2.2.6 Cytoskeletal protein: Vimentin

Vimentin is a type III intermediate filament that play a functional role for cellular organization, organelle distribution, and EMT (Hwang and Ise, 2020; Viedma-Poyatos et al., 2020). HSC activation manifested as proliferation and migration is an important event involved in the progression of liver fibrosis. Vimentin is highly expressed with desmin during HSC activation (Zhang et al., 2018). It contributes to stabilizing focal adhesion, which governs cell migration and motility (Tsuruta and Friedman, 2017).

## 5 NPCs: Kupffer cells

Kupffer cells are liver-resident macrophages that play a critical role in the innate immune response (Wen et al., 2021). Kupffer cells have high heterogeneity and plasticity, allowing them to maintain homeostasis and perform defense functions (Wen et al., 2021). They are the most abundant macrophage, accounting for about 80%–90% of total macrophages in mammalian bodies (Chaudhry et al., 2019). Hepatic macrophages are mainly composed of Kupffer cells originating from fetal yolk sacs and monocytes/macrophages derived from bone marrow (Beattie et al., 2016). Kupffer cells are the first innate immune cells that exert a protective response against infection by pathogens in the liver (Li et al., 2017; Chen et al., 2020). They have self-renewal capacity and can differentiate into classically activated M1 macrophages or alternatively activated M2 macrophages depending on microenvironmental signals (Li et al., 2017; Chen et al., 2020). Proinflammatory M1 macrophages are induced by LPS or interferon gamma (IFNγ) and produce huge amount of inflammatory cytokines. They exhibit a strong ability to present antigens and elevated nitric oxide and ROS production (Tan et al., 2016), and also show pro-glycolytic activity to increase energy efficiency and availability in the hypoxic state (Tawakol et al., 2015; Chen et al., 2022). In addition, they are involved in directing T-cells toward the type 1 T helper cell phenotype. Conversely, M2 macrophages exhibit anti-inflammatory properties that can counterbalance proinflammatory M1 macrophages by releasing anti-inflammatory cytokines to reduce inflammatory activity (Ruytinx et al., 2018). These cells play a key role in synthesizing essential mediators associated with tissue remodeling and angiogenesis, and enhance T helper cell type 2 immune responses. In normal conditions, M2-type cells stimulate the apoptosis of M1-type cells to maintain the M1/M2 balance, which is considered to play an important role in maintaining liver homeostasis and liver function (Wan et al., 2014).

**FIGURE 6:**
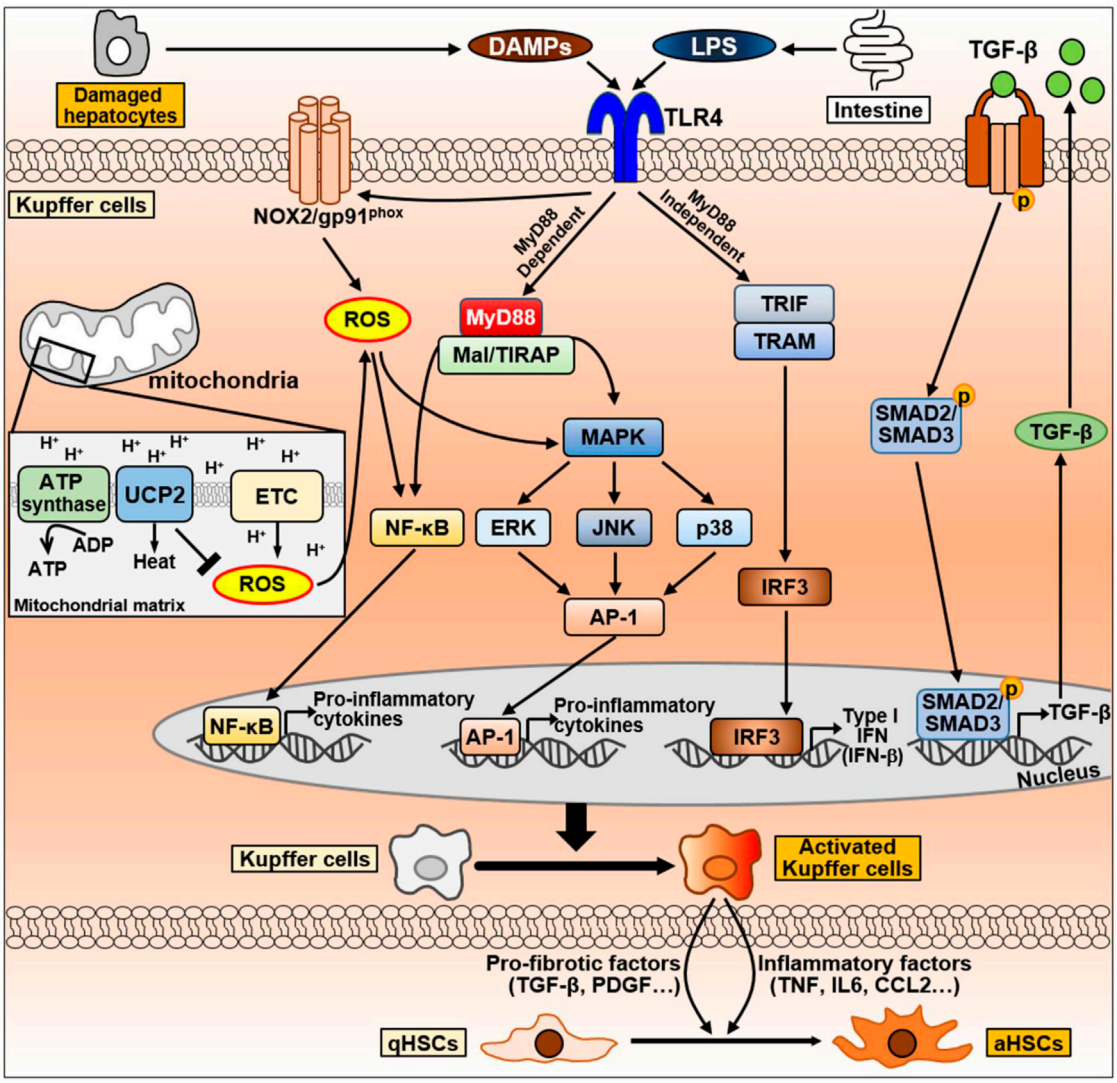
The signaling pathway of Kupffer cells during NAFLD progression. Kupffer cells can be activated by DAMPs released from damaged hepatocytes and LPS secreted by intestine during NAFLD progression. DAMPs and LPS bind to TLR4 on Kupffer cell membrane and activates TLR4 signaling. TLR4-mediate signaling pathways in Kupffer cells are divided into MyD88-dependent or MyD88-independent signaling. MyD88-dependent signaling is mediated by MyD88 and Mal/TIRAP, which activate MAPK signaling, including ERK, JNK, and p38, and activate NF-κB and AP-1 to produce pro-inflammatory cytokines. On the other hand, the MyD88-independent signaling is mediated by TRIF and TRAM, leading to the activation of IRF3 and regulates the expression of IFN. LPS can also promote NOX2 to produce ROS via TLR4 activation. Excessive ROS production by mitochondria promotes NF-κB and MAPK signaling to secrete pro-inflammatory cytokines. Conversely, UCP2, a mitochondrial inner membrane protein, counteracts ROS production to maintain homeostasis. Abbreviation: AP-1, activator protein 1; ETC., mitochondrial electron transport chain; IRF3, interferon regulatory factor 3; Mal/TIRAP, MyD88 adaptor-like/TIR domain containing adaptor protein; MAPK, mitogen-activated protein kinase; MyD88, myeloid differentiation primary response 88; NF-κB, Nuclear factor kappa B; NOX2, NADPH oxidase 2; TRAM, TRIF adaptor-related adaptor molecule; TRIF, Toll/IL-1R domain-containing adaptor-inducing IFN-β; ROS, Reactive oxygen species; UCP2, uncoupling protein 2.

In NASH progression, Kupffer cells are the first cells to respond to the liver microenvironment, and they have critical importance in accelerating NASH (Chen et al., 2020). Hepatic steatosis alters the M1/M2 phenotypic balance through various signals, which evolves into chronic inflammation of the liver (Lefere and Tacke, 2019). In addition, the ambivalence of activated Kupffer cells contributes to NASH progression. For example, in NASH, M2 type Kupffer cells have the advantage of reducing hepatic inflammation by inhibiting the activation of M1 type Kupffer cells, but have the ability to induce fibrosis (Qian et al., 2020). Understanding the intracellular signal to determine the Kupffer cell phenotype and its effects on other cells or organs could be a great help in the development of NASH treatments. Additionally, the diverse population of hepatic macrophages exhibits different phenotypes and distinct behaviors. Therefore, there is a need to understand the complex heterogeneity and functional diversity of macrophages in the liver. In this section, we discussed the signaling pathways in Kupffer cells during NAFLD progression and summarized in Figure 6.

### 5.1 Cellular plasticity in Kupffer cells during NAFLD progression

#### 5.1.1 Toll-like receptor 4 (TLR4) pathway in Kupffer cells

TLR4 is a transmembrane protein member of the TLR family that mediates the activation of innate immune responses and recognizes LPS (Kawasaki and Kawai, 2014). Obese individuals or patients with NAFLD have an overgrowth of intestinal bacteria, resulting in increased LPS production compared to the findings in normal-weight individuals (Wigg et al., 2001; Crane et al., 2015). Kupffer cells express TLR4, which recognizes LPS. Based on the gut–liver axis, intestine-derived LPS activate Kupffer cells by binding to TLR4. Activation of TLR4-mediated signaling pathway triggers large amounts of pro-inflammatory cytokines through nuclear factor-kappa B (NF-κB), MAPK, extracellular signal-regulated kinase 1, p38, JNK, and interferon regulatory factor 3 (IRF3) (Bieghs and Trautwein, 2013). Consequently, excessive pro-inflammatory cytokines can be produced during liver fibrosis, and they cause liver damage through the activation of HSCs (Bieghs and Trautwein, 2013).

TLR4 signaling consists of myeloid differentiation primary response 88 [MyD88]-dependent and MyD88-independent signaling pathways, which are mediated by various adaptor proteins. The MyD88-dependent pathway induces pro-inflammatory cytokines by directly stimulating NF-κB and activator protein 1 (AP-1) through the adaptor molecules MyD88 and MyD88 adaptor-like (Mal, also termed TIRAP) (Akira and Takeda, 2004; Guo and Friedman, 2010). On the other hand, the MyD88-independent pathway is mediated by Toll/IL-1R domain-containing adaptor-inducing IFN-β (TRIF) and TRIF adaptor-related adaptor molecule (TRAM) (Kawai et al., 2001; Doyle et al., 2002; Yamamoto et al., 2003a; Yamamoto et al., 2003b; Kagan et al., 2008; Tanimura et al., 2008). These adaptors activate IRF3, which regulates type I IFN expression, late NF-κB activation, and pro-inflammatory immune responses (Kawai et al., 2001; Doyle et al., 2002; Akira and Takeda, 2004). Activated Kupffer cells produce inflammatory factors including TNF and IL-6 and recruit infiltrating inflammatory cells. It is thought that these events contribute to NAFLD progression at multiple levels (Leroux et al., 2012). As endogenous TLR4 ligands, DAMPs derived from damaged hepatocytes can stimulate Kupffer cells (Cha et al., 2018; Takimoto et al., 2023). DAMPs can act on TLR4, which activates M1-type Kupffer cell and promote the secretion of pro-inflammatory cytokines (Takimoto et al., 2023).

#### 5.1.2 ROS-related signaling pathways in Kupffer cells

ROS critically affect Kupffer cell function. The primary source of ROS in macrophages is NOX (Canton et al., 2021). Kupffer cells generate superoxide, a major form of ROS, through the phagocytic NOX2 in response to microbial stimuli, including LPS (Lambeth, 2004). It helps kill microorganisms or stimulates redox-sensitive targets including protein kinase C, NF-κB, and ERK family members (Forman and Torres, 2002).

Mitochondria represent the largest source of metabolic ROS induction (Turrens, 2003). Mitochondrial substrate oxidation creates the electrochemical proton gradient across the inner mitochondrial membrane. The electrochemical energy is used for the synthesis of ATP via oxidative phosphorylation. The energy of the electrochemical gradient can also be dispersed as heat via uncoupling. Among uncoupling proteins, uncoupling protein 2 (UCP2) is distributed in most tissue, and it is abundantly expressed in immune cells (Brand and Esteves, 2005; Mattiasson and Sullivan, 2006). UCP2 overexpression reduces ROS production and immune cell activation (Kizaki et al., 2002; Ryu et al., 2004), whereas loss of UCP2 promotes ROS production, pro-inflammatory cytokine secretion, and NF-κB activation (Arsenijevic et al., 2000; Bai et al., 2005). LPS can inhibit UCP2 expression in macrophages (Arsenijevic et al., 2000; Kizaki et al., 2002). This indicates that TLR4-mediated signaling might use mitochondrial ROS in an amplifying circuit. In fact, LPS-induced TLR4 signaling activates JNK and p38 through mitochondrial ROS in the peritoneal macrophages from ucp2^−/−^ mice (Emre et al., 2007). These findings suggest that UCP2 can inhibit ROS-sensitive TLR4 signaling, including JNK, p38, and NF-κB. ROS-mediated amplification of TLR4 signaling might result from dysfunctional UCP2 in Kupffer cells.

#### 5.1.3 TGF-β pathway in Kupffer cells

Inflammation is a key driver of liver fibrosis (Seki and Schwabe, 2015; Koyama and Brenner, 2017). During liver injury, immune cells, including macrophages, lymphocytes, and eosinophils, infiltrate the damaged area. Lymphocytes generate cytokines and chemokines that activate macrophages (Tanaka and Miyajima, 2016). Activated macrophages stimulate inflammatory cells to secrete pro-inflammatory cytokines and excessively activate inflammatory signals to generate ROS. In fibrosis, macrophages generate fibrosis-promoting factors such as TGF-β and PDGF, and they are found near the myofibroblasts that produce collagen (Seki and Schwabe, 2015; Koyama and Brenner, 2017). These finding suggest that macrophages are closely associated with the activation of myofibroblasts.

Macrophages exhibit a heterogeneous cell population with enormous cellular plasticity and various microenvironmental stimuli cause them to polarize into different phenotypes (Zhu et al., 2015; Viola et al., 2019). Hepatic macrophages consist of Kupffer cells (liver-resident macrophages) and circulating monocytes (inflammatory recruiting macrophages) (Beattie et al., 2016). Both cells can activate HSCs, and they can be transdifferentiated by TGF-β. Resident hepatic macrophages recruit monocytes to promote liver fibrosis by secreting the chemokine CCL2 (Cai et al., 2018; Wen et al., 2021).

Macrophages are classified into pro-inflammatory M1 type and anti-inflammatory M2 type (Chun and Kim, 2018; Yu et al., 2018). M1 macrophages are predominant during liver injury and promote EMT and ECM deposition, whereas M2 macrophages release anti-inflammatory factors including IL-10, arginase, and HO-1 (Li et al., 2017; Chen et al., 2020). However, during chronic liver injury, M2 macrophages can contribute to the production of pro-fibrotic factors, especially TGF-β and PDGF (Sun et al., 2017).

Thus, macrophages are the primary sources of TGF-β, and they are important contributors to the development of liver fibrosis (Seki and Schwabe, 2015; Koyama and Brenner, 2017). TGF-β can cause macrophage polarization towards a M2-like phenotype through SNAIL1 (Zhang et al., 2016). SNAIL overexpression in human THP-1 macrophages stimulates the expression of M2 markers (such as CD206) and anti-inflammatory IL-10. Conversely, SNAIL deficiency triggers M1 polarization by enhancing pro-inflammatory cytokines.

## 6 Conclusion and prospects

Currently, there are no approved therapies for NAFLD despite ongoing attempts. To find an appropriate treatment for NAFLD, it is necessary to observe various factors from a broader perspective. The heterogeneity of NAFLD, including its wide spectrum of clinical and histological characteristics, is a primary factor complicating the treatment of NAFLD. The term NAFLD does not fully reflect current knowledge about this complex disease, which has a complex etiology associated with various metabolic disorders. The introduction of the term MAFLD is an effort to explain the complex pathophysiological mechanism and heterogeneity of NAFLD with several clinical and histological characteristics.

The heterogeneity of NAFLD is closely associated with the cellular heterogeneity and plasticity of cell populations with specialized functions according to their localization in the liver. Under normal conditions, cellular heterogeneity and plasticity in the liver play critical roles in maintaining metabolic homeostasis, adaptation, and defense against pathogens. However, this also endows cells with strong and flexible adaptability to environmental changes. Therefore, in order to adapt to various environmental changes in a complex disease state, these liver cells will undergo complex and diverse changes and will be developed to act in new roles. Although not addressed in this review, understanding cell-to-cell communication as these cells acquire new identities and undergo phenotypic changes is also considered crucial. Therefore, an in-depth understanding of exosomes and cytokines secreted from damaged organs and perspectives on their impact during NAFLD progression will be helpful for developing effective therapeutics for the treatment of heterogeneous and complex NAFLD.
