# Corrigendum: Cellular heterogeneity and plasticity during NAFLD progression

**DOI:** 10.3389/fmolb.2024.1511947

**Published:** 2024-10-28

**Authors:** Hyun-Ju Park, Juyong Choi, Hyunmi Kim, Da-Yeon Yang, Tae Hyeon An, Eun-Woo Lee, Baek-Soo Han, Sang Chul Lee, Won Kon Kim, Kwang-Hee Bae, Kyoung-Jin Oh

**Affiliations:** ^1^ Metabolic Regulation Research Center, Korea Research Institute of Bioscience and Biotechnology (KRIBB), Daejeon, Republic of Korea; ^2^ Department of Functional Genomics, KRIBB School of Bioscience, University of Science and Technology (UST), Daejeon, Republic of Korea; ^3^ Biodefense Research Center, Korea Research Institute of Bioscience and Biotechnology (KRIBB), Daejeon, Republic of Korea

**Keywords:** NAFLD, heterogeneity, hepatocytes, Cholangiocytes, hepatic stellate cells, Kupffer cells, cellular plasticity, metabolic adaptation

In the published article, an **Author** name was incorrectly written as Juyoung Choi. The correct spelling is Juyong Choi.

The authors apologize for this error and state that this does not change the scientific conclusions of the article in any way. The original article has been updated.

